# Diet-induced obesity impairs spermatogenesis: the critical role of NLRP3 in Sertoli cells

**DOI:** 10.1186/s41232-022-00203-z

**Published:** 2022-08-02

**Authors:** Yang Mu, Tai-lang Yin, Yan Zhang, Jing Yang, Yan-ting Wu

**Affiliations:** 1 Reproductive Medicine Center, Renmin Hospital of Wuhan University, Wuhan, 430060 China; 2 Department of Clinical Laboratory, Renmin Hospital of Wuhan University, Wuhan, 430060 China; 3 Institute of Reproduction and Development, Obstetrics and Gynecology Hospital, Fudan University, Shanghai, 200011 China

**Keywords:** AMPKα, MicroRNA, NLRP3, Obesity, Sertoli cells

## Abstract

**Background:**

Accumulating evidence indicates a key role of Sertoli cell (SC) malfunction in spermatogenesis impairment induced by obesity. Nucleotide-binding oligomerization domain-like receptor with a pyrin domain 3 (NLRP3) is expressed in SCs, but the role of NLRP3 in the pathological process of obesity-induced male infertility remains unclear.

**Methods:**

NLRP3-deficient mice were fed a high-fat diet for 24 weeks to establish obesity-related spermatogenesis impairment. In another set of experiments, a lentiviral vector containing a microRNA (miR)-451 inhibitor was injected into AMP-activated protein kinase α (AMPKα)-deficient mouse seminiferous tubules. Human testis samples were obtained by testicular puncture from men with obstructive azoospermia whose samples exhibited histologically normal spermatogenesis. Isolated human SCs were treated with palmitic acid (PA) to mimic obesity model in vitro.

**Results:**

Increased NLRP3 expression was observed in the testes of obese rodents. NLRP3 was also upregulated in PA-treated human SCs. NLRP3 deficiency attenuated obesity-related male infertility. SC-derived NLRP3 promoted interleukin-1β (IL-1β) secretion to impair testosterone synthesis and sperm performance and increased matrix metalloproteinase-8 (MMP-8) expression to degrade occludin via activation of nuclear factor-kappa B (NF-κB). Increased miR-451 caused by obesity, decreased AMPKα expression and sequentially increased NADPH oxidase activity were responsible for the activation of NLRP3. miR-451 inhibition protected against obesity-related male infertility, and these protective effects were abolished by AMPKα deficiency in mice.

**Conclusions:**

NLRP3 promoted obesity-related spermatogenesis impairment. Increased miR-451 expression, impaired AMPKα pathway and the subsequent ROS production were responsible for NLRP3 activation. Our study provides new insight into the mechanisms underlying obesity-associated male infertility.

**Supplementary Information:**

The online version contains supplementary material available at 10.1186/s41232-022-00203-z.

## Background

Infertility now affects approximately 15% of families worldwide [1]. Male factors are known to contribute to 25–30% of infertility cases, and obesity is one of the risk factors for male infertility [2–4]. Spermatogenesis impairment induced by obesity is multifactorial and includes endocrine disorders, genetic components, and chemical factors [5–7]. Recently, accumulating evidence has indicated a key role of Sertoli cell malfunction in spermatogenesis [8, 9].

Sertoli cells (SCs) exert an important role in the development of testes and spermatogenesis, as they provide structural and nutritional support to germ cells [10]. Tight junctions between the basolateral membranes of adjacent SCs form a blood-testis barrier (BTB) in the seminiferous epithelium, protecting germ cells from internal antigens and exogenous toxic chemical attack [11, 12]. The number of SCs defines and predicts the population size of germ and Leydig cells in adult mice [13]. In addition, SCs modulate testosterone production by cytokine secretion [14, 15]. Previous studies have revealed that obesity affects spermatogenesis by impairing the BTB [16–18]. However, the precise role of SCs in obesity-related impairment of spermatogenesis remains unclear.

Nucleotide-binding oligomerization domain-like receptor with a pyrin domain 3 (NLRP3) inflammasome is the most studied inflammasome and comprises NLRP3, apoptosis-associated speck-like protein (ASC), and pro-caspase-1 [19]. NLRP3 has been regarded as a sensor of cellular damage. Once activated, NLRP3 recruits ASCs to form a caspase-1-activating platform and induces the secretion of interleukin-1β (IL-1β) and IL-18, thus creating an inflammatory microenvironment [20]. Activation of NLRP3 promoted the development of uropathogenic *Escherichia coli*-induced orchitis [21]. Recently, NLRP3 was found to be expressed in SCs [22]. Increased NLRP3 expression was also observed in the testes of obese mice [23]. These observations raised the possibility that NLRP3 is involved in the pathological process of obesity-induced SC dysfunction and male infertility.

In the study reported here, we found that NLRP3 was upregulated in testicular SCs. NLRP3 deficiency attenuated obesity-induced SC damage and BTB impairment, improving testosterone secretion by Leydig cells and sperm quality. We also found that decreased AMP-activated protein kinase α (AMPKα) expression and the subsequently increased NADPH oxidase activity were responsible for the activation of NLRP3 in the SCs of obese mice. These data advance our understanding of SCs in obesity-induced spermatogenesis impairment.

## Results

### NLRP3 was upregulated in SCs isolated from the testes of obese mice

**Fig. 1:**
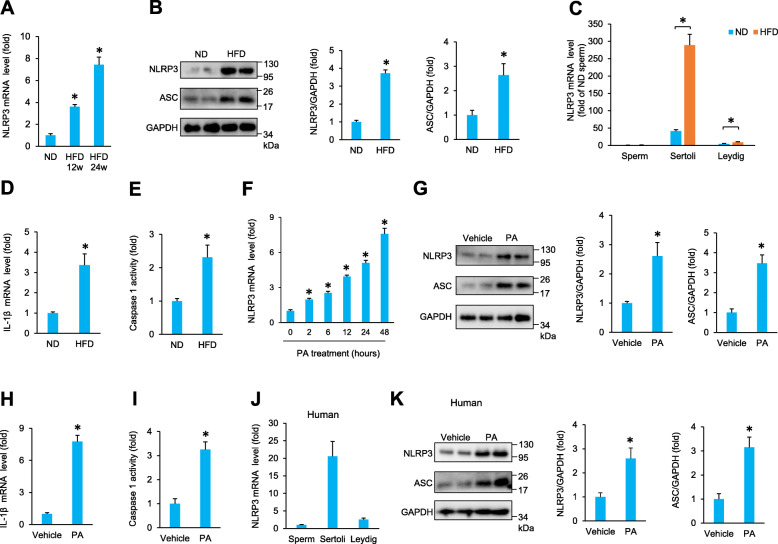
NLRP3 expression is upregulated by obesity. **A** The mRNA levels of NLRP3 in the testes of obese mice (*n* = 6). **B** Western blot analysis and quantitative results of NLRP3 and ASC in the testes of obese mice (*n* = 6). (C) NLRP3 mRNA levels in cell populations isolated from the testes of obese mice (*n* = 6). **D** The mRNA level of IL-1β in primary SCs isolated from HFD mice (*n* = 6). **E** Caspase 1 activity in primary SCs isolated from HFD mice (*n* = 6). **F** The mRNA levels of NLRP3 in primary SCs (*n* = 6). **G** Western blot analysis and quantitative results of NLRP3 and ASC in primary SCs (*n* = 6). **H**, **I** The mRNA levels of IL-1β and caspase 1 activity in primary SCs (*n* = 6). **J** NLRP3 mRNA levels in cell populations isolated from the human testis (*n* = 5). **K** Western blot analysis and quantitative results of NLRP3 and ASC in human primary SCs (*n* = 5). Data are presented as the mean ± SD. For **A** and **F**, the statistical analysis was carried out by Student’s two-tailed *t* test; for others, the statistical analysis was carried out by one-way ANOVA. *P < 0.05 vs the matched control

To investigate the potential role of NLRP3 in the pathological processes of obesity-related impairment of spermatogenesis, we first examined whether the NLRP3 expression levels were altered in the testes of obese mice. The mRNA levels of NLRP3 were increased in the testes of mice fed a high-fat diet (HFD) (Fig. 1A). Western blotting showed that NLRP3 and downstream ASC were dramatically upregulated in the testes of obese mice compared with those of normal diet (ND)-fed mice (Fig. 1B). To determine the cellular source of NLRP3 in these diseased testes, we isolated sperm, SCs and Leydig cells from mouse testes at 24 weeks after ND or HFD. In ND-treated mice, the NLRP3 mRNA levels were much higher in SCs than in sperm and Leydig cells (Fig. 1C). Surprisingly, the HFD had no significant effect on NLRP3 mRNA in sperm, slightly increased the NLRP3 mRNA levels in Leydig cells, and markedly increased the NLRP3 mRNA levels in SCs (Fig. 1C), suggesting that NLRP3 is mainly derived from SCs under obesity conditions. Further detection revealed that the IL-1β mRNA levels and caspase 1 activity were increased in primary SCs isolated from HFD mice (Fig. 1D, E). Next, the isolated SCs were subjected to palmitic acid (PA) to mimic the in vivo microenvironment of obesity. In addition, NLRP3 upregulation was also found in isolated SCs that were stimulated with PA for 2, 6, 12, 24, or 48 h (Fig. 1F). In line with this finding, NLRP3 and ASC protein expression was also increased in response to PA treatment (Fig. 1G). PA treatment also increased the IL-1β mRNA levels and caspase-1 activity in primary SCs (Fig. 1H, I). Next, we detected NLRP3 in human testis samples. Human testis samples were obtained by testicular puncture from men with obstructive azoospermia whose samples exhibited histologically normal spermatogenesis. The patient characteristics are summarized in Table 1. Similar to the data in mice, NLRP3 was mainly expressed in human SCs (Fig. 1J). We found that NLRP3 and downstream ASC were also upregulated in human SCs in response to PA (Fig. 1K).

**Table 1:** Patient characteristics and sperm parameters

Patient no.	#1	#2	#3	#4	#5
Age (years)	24	32	25	36	30
BMI (kg/m^2^)	22.43	18.97	19.54	22.45	23.54
Testosterone (ng/mL)	8.11	5.03	6.46	9.32	5.62
FSH (mIU/mL)	3.24	2.14	3.26	4.25	2.87

Human testis samples were obtained by testicular puncture from men with obstructive azoospermia whose samples exhibited histologically normal spermatogenesis

### NLRP3 deficiency increased the expression of ZO-1 and occludin in PA-treated TM4 cells

**Fig. 2:**
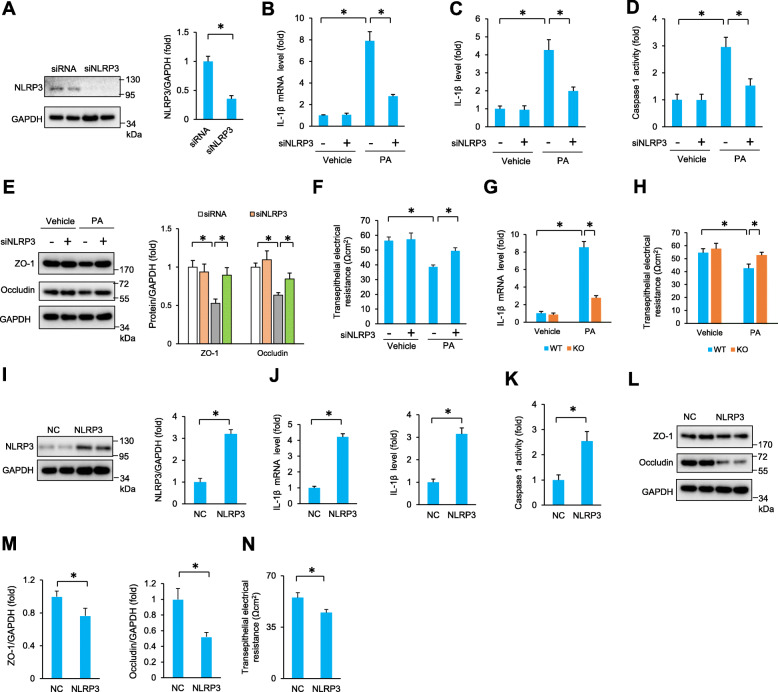
NLRP3 deficiency attenuated palmitic acid-induced impairment of SCs in vitro. **A** NLRP3 expression was detected in TM4 cells at 24 h after siNLRP3 infection (*n* = 6). **B**, **C** The IL-1β mRNA and protein expression in TM4 cells after PA incubation for 24 h (*n* = 6). **D** Caspase 1 activity in TM4 cells after PA incubation for 24 h (*n* = 6). **E** The protein expression of ZO-1 and occludin in TM4 cells after PA incubation for 24 h (*n* = 6). **F** Transepithelial electrical resistance was detected in TM4 cells after PA incubation for 48 h (*n* = 6). **G** The IL-1β mRNA in primary SCs isolated from NLRP3-deficient mice (*n* = 4–5). **H** Transepithelial electrical resistance was detected in primary SCs isolated from NLRP3-deficient mice (*n* = 4–5). **I** NLRP3 expression was detected in TM4 cells at 48 h after NLRP3 overexpression (*n* = 6). **J** The IL-1β mRNA and protein expression in TM4 cells at 48 h after NLRP3 overexpression (*n* = 6). **K** Caspase 1 activity in TM4 cells at 48 h after NLRP3 overexpression (*n* = 6). **L**, **M** The protein expression of ZO-1 and occludin in TM4 cells at 48 h after NLRP3 overexpression (*n* = 6). **N** Transepithelial electrical resistance was detected in TM4 cells at 48 h after NLRP3 overexpression (*n* = 6). Data are presented as the mean ± SD. For **A** and **I**–**N**, the statistical analysis was carried out by Student’s two-tailed *t* test; for others, the statistical analysis was carried out by one-way ANOVA. *P < 0.05 vs the matched control

The dramatic increase in NLRP3 expression in obese testes prompted us to investigate whether the increased NLRP3 contributed to PA-induced TM4 dysfunction. We knocked down NLRP3 expression in TM4 cells by infection with siRNA targeting NLRP3 (siNLRP3). Infection with siNLRP3 resulted in a decrease in NLRP3 expression, as determined by Western blotting (Fig. 2A). NLRP3 silencing significantly suppressed the elevation of IL-1β levels and caspase 1 activity in response to PA treatment (Fig. 2B–D). Several integral membrane proteins, including occludin and ZO-1, have been reported to play key roles in maintaining the integrity of tight junctions [11]. Moreover, some reproductive toxicants could target these proteins, thus impairing the BTB and resulting in spermatogenic dysfunction [24]. Therefore, we detected alterations in occludin and ZO-1. As expected, NLRP3 knockdown reversed occludin and ZO-1 degradation in TM4 cells exposed to PA (Fig. 2E). Next, we used transepithelial electrical resistance to detect the integrity of tight junctions and found that PA-induced impairment of the integrity of tight junctions was attenuated by siNLRP3 infection (Fig. 2F). We also use SCs isolated from NLRP3-deficient mice, and found that NLRP3 knockout inhibited the elevation of IL-1β levels in response to PA treatment (Fig. 2G). PA-induced impairment of the integrity of tight junctions was also attenuated in NLRP3-deficienct SCs when compared with that in SCs isolated from wild-type (WT) control (Fig. 2H). To further confirm the role of NLRP3, TM4 cells were infected with adenovirus carrying NLRP3 (Fig. 2I). This overexpression of NLRP3 led to the elevation of IL-1β levels and caspase 1 activity as well as a decrease in ZO-1 and occludin expression (Fig. 2J–M). NLRP3 overexpression alone impaired the integrity of tight junctions in TM4 cells (Fig. 2N).

### NLRP3 regulated the expression of occludin via NF-κB/MMP-8 in vitro

**Fig. 3:**
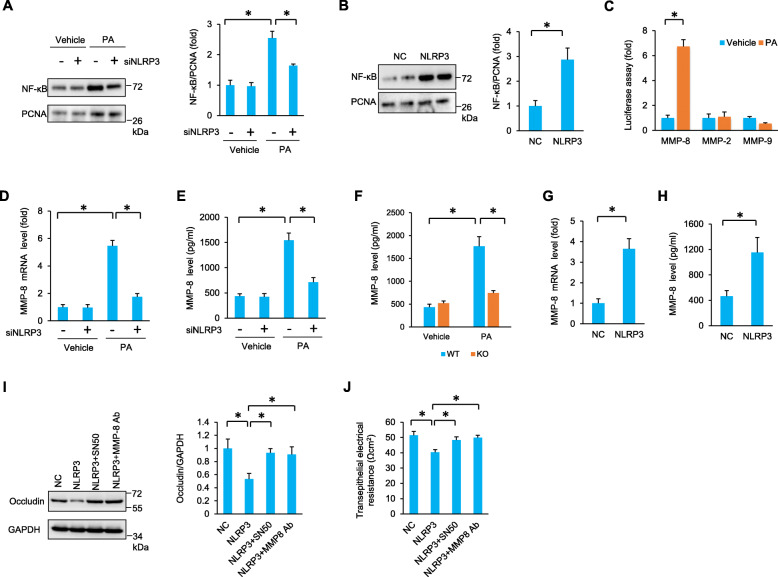
NLRP3 regulated the expression of occludin via NF-κB/MMP-8 in vitro. **A** The nuclear accumulation of NF-κB was detected in TM4 cells after PA incubation for 24 h (*n* = 6). **B** The nuclear accumulation of NF-κB was detected in TM4 cells at 48 h after NLRP3 overexpression (*n* = 6). **C** The luciferase assay revealed the binding of NF-κB to the promoter of MMP-8 in TM4 cells after PA incubation for 24 h (*n* = 6). **D**–**E** MMP-8 mRNA and expression in TM4 cells after PA incubation for 24 h (*n* = 6). **F** MMP-8 expression in isolated NLRP3-deficient SCs after PA incubation for 24 h (*n* = 4–5). **G**, **H** MMP-8 mRNA and expression in TM4 cells after NLRP3 overexpression (*n* = 6). **I** The protein expression of occludin in TM4 cells at 48 h after SN50 or MMP-8 blocking peptide incubation (*n* = 6). **J** Transepithelial electrical resistance was detected in TM4 cells at 48 h after SN50 or MMP-8 blocking peptide incubation (*n* = 6). Data are presented as the mean ± SD. For **B, C** and **G**, **H**, the statistical analysis was carried out by Student’s two-tailed *t* test; for others, statistical analysis was carried out by one-way ANOVA. *P < 0.05 vs the matched control

NLRP3 regulates the nuclear factor-kappa B (NF-κB) signalling pathway, and NF-κB might mediate the downstream effects of NLRP3 in mice [25]. Next, we investigated whether NLRP3 regulated the expression of tight junction proteins via the NF-κB signalling pathway. We found that PA-induced nuclear accumulation of NF-κB was attenuated by NLRP3 deficiency in vitro (Fig. 3A). NLRP3-overexpressing TM4 cells displayed the opposite pattern, and NLRP3 overexpression led to spontaneous activation of NF-κB (Fig. 3B). We also found that NLRP3-medicated NF-κB activation was blocked by TM4 culture medium-specific depletion of IL-1β with a mouse IL-1β neutralizing antibody (Figure S1). NF-κB is crucial for transactivation of matrix metalloproteinases (MMPs) [9, 26, 27]. The luciferase assay revealed that PA treatment promoted the binding of NF-κB to the MMP-8 promoter but not to the MMP-2 or MMP-9 promotor (Fig. 3C). We also found that NLRP3 deficiency significantly reduced the elevation of MMP-8 mRNA (Fig. 3D), and NLRP3 deficiency decreased the release of MMP-8 to the medium from PA-treated cells (Fig. 3E). Decreased MMP-8 expression was also observed in NLRP3-deficient primary SCs when compared with that in primary SCs isolated from WT mice (Fig. 3F). Further detection also revealed that NLRP3 overexpression increased the MMP-8 mRNA levels and promoted MMP-8 secretion (Fig. 3G, H). To confirm the role of NF-κB and MMP-8 in NLRP3-mediated occludin degradation, TM4 cells were subjected to an NF-κB inhibitor (SN50) or an MMP-8 blocking peptide. The data in our study suggested that NLRP3-mediated occludin degradation was blocked by NF-κB or MMP-8 blockade (Fig. 3I). The integrity of tight junctions, as detected by transepithelial electrical resistance, was decreased in response to PA treatment but increased by SN50 treatment or an MMP-8 blocking peptide (Fig. 3J).

### NLRP3 affected Leydig cells and sperm in a paracrine-dependent manner

To exclude the direct role of NLRP3 in PA-induced dysfunction of Leydig cells, we first knocked down NLRP3 in Leydig cells. As shown in Figure S2A, PA decreased testosterone production, and NLRP3 deficiency did not affect the production of testosterone. Interestingly, we found that IL-1β alone significantly decreased the production of testosterone in TM3 cells (Figure S2B). Given that IL-1β has a pronounced effect on the production of testosterone, we hypothesized that IL-1β secretion from SCs with NLRP3 overexpression might suppress testosterone synthesis. The conditioned medium of NLRP3-overexpressing TM4 cells was cocultured with TM3 cells. Testosterone production was shown to be decreased in TM3 cells after they were cocultured with conditioned medium from NLRP3-overexpressing TM4 cells. This hypothesis was further supported by using a mouse IL-1β neutralizing antibody to block IL-1β activity. Interestingly, we found that after IL-1β neutralization, there was no difference in testosterone production between the TM3 cells cocultured with conditioned medium of NLRP3-overexpressing TM4 cells and the TM3 cells cocultured with conditioned medium of TM4 cells with negative control (NC) (Figure S2C). Next, we detected the mRNA levels of steroidogenic enzymes, including steroidogenic acute regulatory (StAR), cholesterol side-chain cleavage P450 (P450scc), 3β-hydroxysteroid dehydrogenase-14-15 isomerase (3β-HSD), 17α-hydroxylase/C17-20 lyase (P450c17), and 17β-hydroxysteroid dehydrogenase (17β-HSD). The data in our study showed that the StAR mRNA levels were increased, whereas the mRNA levels of P450scc and P450c17 were decreased, and no significant differences were found in the expression of 3β-HSD or 17β-HSD (Figure S2D). These pathological alterations were abolished by the use of this mouse IL-1β neutralizing antibody. In line with the findings in TM3 cells, sperm viability, and motility were decreased after PA treatment, but the two were not affected by NLRP3 inhibition (Figure S3A-B). IL-1β treatment decreased sperm viability and motility (Figure S3C-D). The conditioned medium collected from NLRP3-overexpressing TM4 cells impaired sperm viability and motility, and these effects were prevented by the use of this mouse IL-1β neutralizing antibody (Figure S3E-F). Taken together, these findings indicated that IL-1β secretion from SCs impaired the function of Leydig cells and sperm in a paracrine-dependent manner.

### NLRP3 deletion improved HFD-induced male infertility

**Fig. 4:**
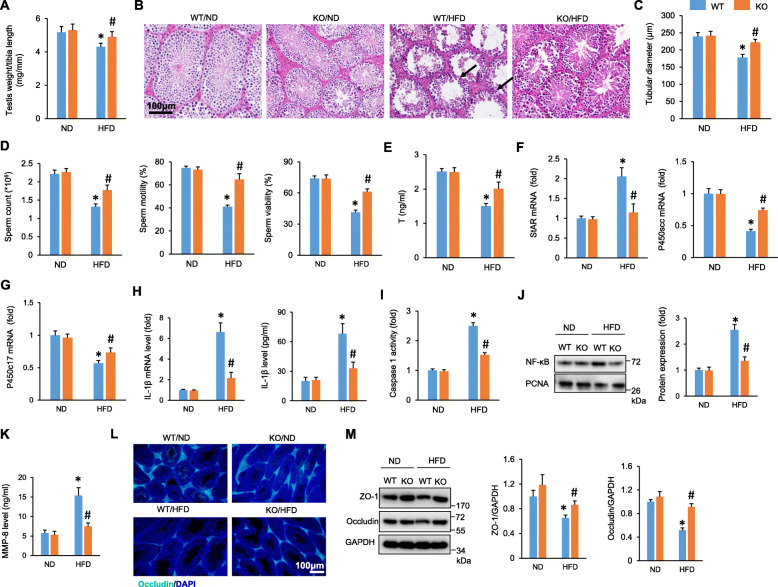
NLRP3 deficiency protected against HFD-induced subfertility in male mice. **A** Statistical results of the ratio of testis weight and tibia length in the indicated groups (*n* = 12). **B**, **C** HE staining of testes and tubular diameter from the indicated groups (*n* = 6). Black arrows indicate destruction of the BTB. **D** Sperm count, viability, and motility of the indicated groups (*n* = 6). **E** Serum testosterone concentration of the indicated groups (*n* = 6). **F**, **G** The mRNA of steroidogenic enzymes in the testes of obese mice (*n* = 6). **H** The expression of IL-1β in the testes of obese mice (*n* = 6). **I** Caspase 1 activity in the testes of obese mice (*n* = 6). **J** The nuclear accumulation of NF-κB was detected in the testes of obese mice (*n* = 6). **K** MMP-8 expression in the testes of obese mice (*n* = 6). **L** Immunofluorescence of occludin in the testes of obese mice (*n* = 5). **M** The protein expression of ZO-1 and occludin in the testes of obese mice (*n* = 6). Data are presented as the mean ± SD. Statistical analysis was carried out by one-way ANOVA. *P < 0.05 vs WT/ND; #*P* < 0.05 vs WT/HFD

To further confirm the role of NLRP3 in obesity-related impairment of spermatogenesis, NLRP3-deficient mice were fed a HFD for 24 weeks. As shown in Fig. 4A, HFD-induced testis atrophy was attenuated by NLRP3 knockout (Fig. 4A). The histological analysis also revealed that HFD-induced destruction of the BTB was improved by NLRP3 knockout in SCs (Fig. 4B). NLRP3 depletion also increased the diameter of the seminiferous tubules in obese mice (Fig. 4C). The sperm count, sperm viability, and sperm motility were decreased by HFD but largely improved by NLRP3 depletion (Fig. 4D). NLRP3 depletion also increased testosterone concentrations in obese mice (Fig. 4E). In response to the HFD, the StAR mRNA levels were increased, whereas the mRNA levels of P450scc and P450c17 were decreased (Fig. 4F, G). However, these pathological alterations were inhibited by NLRP3 deficiency (Fig. 4F, G). In line with the in vitro findings, we also found that the HFD-induced elevation of IL-1β was drastically attenuated in NLRP3-deficient mice (Fig. 4H). Correspondingly, the caspase 1 activity was significantly suppressed in these KO mice compared with testis samples from the WT control (Fig. 4I). Further detection revealed that NLRP3 attenuated the nuclear accumulation of NF-κB in the testes of obese mice (Fig. 4J). The increased MMP-8 in the testes of obese mice was also suppressed by NLRP3 depletion (Fig. 4K). Immunofluorescence analyses revealed that NLRP3 deficiency significantly increased occludin protein expression in the testes of obese mice (Fig. 4L). Next, we detected the expression of ZO-1 and occludin by Western blotting and found that ZO-1 and occludin protein expression was decreased in the testes of obese mice and inhibited by NLRP3 depletion (Fig. 4M).

### HFD-induced ROS generation was responsible for NLRP3 upregulation

**Fig. 5:**
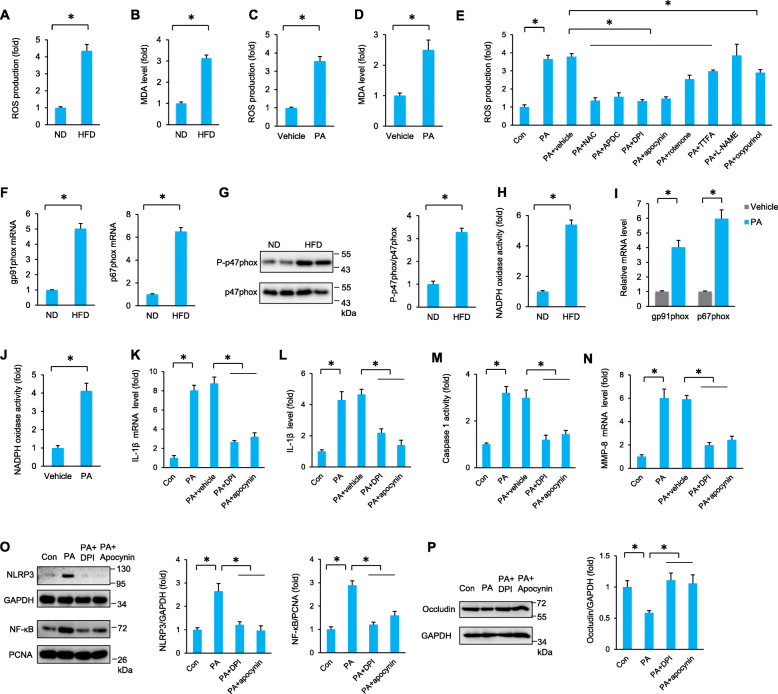
ROS production regulates NLRP3 activation in the testis during obesity. **A**, **B** The ROS production and MDA content in the testes of the indicated groups (*n* = 6). **C**, **D** The ROS production and MDA content in PA-treated TM4 cells (*n* = 6). **E** The ROS production in TM4 cells that were treated with various inhibitors (*n* = 6). **F** The mRNA levels of gp91phox and p67phox in the testes of the indicated groups (*n* = 6). **G** The phosphorylation of p47phox in the testes of the indicated groups (*n* = 6). **H** The activity of NADPH oxidase in the testes of the indicated groups (*n* = 6). **I** The mRNA levels of gp91phox and p67phox in cultured TM4 cells at 24 h after PA incubation (*n* = 6). **J** The activity of NADPH oxidase in PA-treated TM4 cells (*n* = 6). **K**, **L** The expression of IL-1β in PA-treated TM4 cells (*n* = 6). **M** Caspase 1 activity in PA-treated TM4 cells (*n* = 6). **N** The mRNA level of MMP-8 in PA-treated TM4 cells (*n* = 6). **O** Nuclear NF-κB and NLRP3 activation was detected in PA-treated TM4 cells (*n* = 6). **P** The protein expression of occludin in PA-treated TM4 cells (*n* = 6). Data are presented as the mean ± SD. For **A**–**D** and **F**–**J**, the statistical analysis was carried out by Student’s two-tailed *t* test; for others, the statistical analysis was carried out by one-way ANOVA. *P < 0.05 vs the matched control

Next, we investigated the reason for the significant NLRP3 activation in the testis during the HFD. Currently, three models for NLRP3 activation have been proposed: the reactive oxygen species (ROS) model, the K^+^ efflux model, and the lysosome rupture model [28]. The ROS model is the most common model for the activation of NLRP3 [29]. Moreover, ROS scavengers can significantly suppress NLRP3 activation [29]. We next detected the production of ROS in the testis using electron spin resonance spectroscopy. As shown in Fig. 5A, B, ROS production and the malondialdehyde (MDA) levels were increased in the testes of HFD mice. PA treatment also increased ROS production and the MDA content in the TM4 cells (Fig. 5C, D). To reveal the resource of increased cellular ROS, TM4 cells were treated with non-specific ROS inhibitors Nacetyl-L-cysteine (NAC) or (2R,4R)-4-aminopyrrolidine-2,4-dicarboxylate (APDC), the NADPH oxidase inhibitors diphenylene iodonium (DPI) and apocynin, the inhibitor of mitochondrial complex I rotenone and the inhibitor of mitochondrial complex II TTFA, the endothelial nitric oxide synthase inhibitor (L-NAME) and an inhibitor of xanthine oxidase (oxypurinol). The data in this study revealed that non-specific ROS inhibitors (NAC or APDC) and NADPH oxidase inhibitors (DPI or apocynin) had similar inhibitory effects on the production of ROS in vitro (Fig. 5E). The other inhibitors, including rotenone, TTFA, L-NAME, and oxypurinol, showed no significant effect or a slight effect on PA-induced ROS production (Fig. 5E). NADPH oxidase activation requires membrane translocation of the cytoplasmic subunits p47phox and p67phox to a membrane-bound heterodimer cytochrome comprising gp91phox and p22phox [30]. Further detection revealed increased mRNA levels of gp91phox and p67phox, phosphorylation of p47phox and NADPH oxidase activity in the testes of obese mice (Fig. 5F–H). In line with these data, we also found that PA increased the mRNA levels of gp91phox and p67phox as well as the NADPH oxidase activity in cultured TM4 cells (Fig. 5I–J). As expected, DPI and apocynin blocked the elevation in IL-1β and caspase 1 activity in PA-treated TM4 cells (Fig. 5K–M). The increased MMP-8 mRNA level was also suppressed by DPI and apocynin (Fig. 5N). PA-induced nuclear accumulation of NF-κB and NLRP3 upregulation were also blocked by DPI and apocynin treatment (Fig. 5O). PA-induced occludin degradation was also abolished by DPI and apocynin treatment (Fig. 5P).

### Increased ROS production was attributed to the impaired AMPKα signalling pathway

**Fig. 6:**
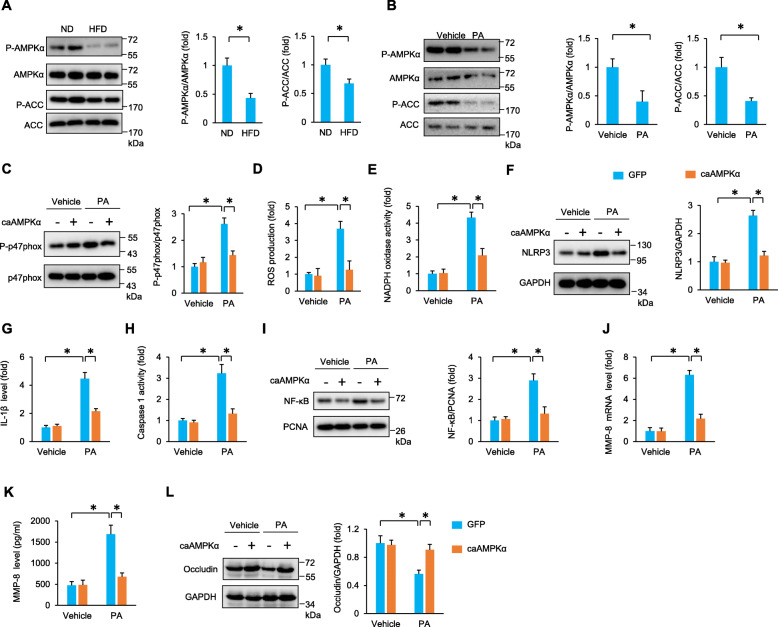
Constitutively active AMPKα attenuated palmitic acid-induced impairment of SCs in vitro. **A**, **B** The protein expression of AMPKα and ACC (*n* = 6). **C** The protein expression of p47phox in PA-treated TM4 cells at 24 h after caAMPKα infection (*n* = 6). **D**, **E** The production of ROS and NADPH oxidase activity in PA-treated TM4 cells at 24 h after caAMPKα infection (*n* = 6). **F**–**H** The NLRP3 and IL-1β levels and caspase 1 activity in PA-treated TM4 cells at 24 h after caAMPKα infection (*n* = 6). **I** The nuclear NF-κB accumulation in PA-treated TM4 cells at 24 h after caAMPKα infection (*n* = 6). **J**, **K** MMP-8 expression in PA-treated TM4 cells at 24 h after caAMPKα infection (*n* = 6). **L** The protein expression of occludin in PA-treated TM4 cells at 24 h after caAMPKα infection (*n* = 6). Data are presented as the mean ± SD. For **A**, **B**, the statistical analysis was carried out by Student’s two-tailed *t* test; for others, the statistical analysis was carried out by one-way ANOVA. *P < 0.05 vs the matched control

AMPKα has been demonstrated to be a negative regulator of ROS production and NLRP3 activation [31]. In our study, we also found that phosphorylated AMPK was significantly decreased in the testes of HFD mice (Fig. 6A). Next, we detected the activity of AMPK, which was reflected by phosphorylated acetyl-CoA carboxylase (ACC). As shown in Fig. 6A, the phosphorylation of ACC was decreased in the testes of obese mice. PA decreased the phosphorylation of AMPK and ACC in vitro (Fig. 6B). To confirm the regulation of ROS production by AMPKα, TM4 cells were subjected to adenoviral infection to overexpress constitutively active AMPKα (Figure S4A). Restoration of AMPKα activation inhibited the phosphorylation of p47phox and reduced ROS production and NADPH oxidase activity in PA-treated TM4 cells (Fig. 6C–E). Restoration of AMPKα activation also suppressed NLRP3 upregulation in response to PA treatment (Fig. 6F). Further detection revealed that AMPKα reactivation decreased IL-1β and caspase 1 activity in response to PA treatment (Fig. 6G, H). PA-induced nuclear accumulation of NF-κB, MMP-8 activation, and subsequent occludin degradation were blocked by caAMPKα infection (Fig. 6I–L).

### miR-451 was responsible for the decreased AMPKα in obese mice

**Fig. 7:**
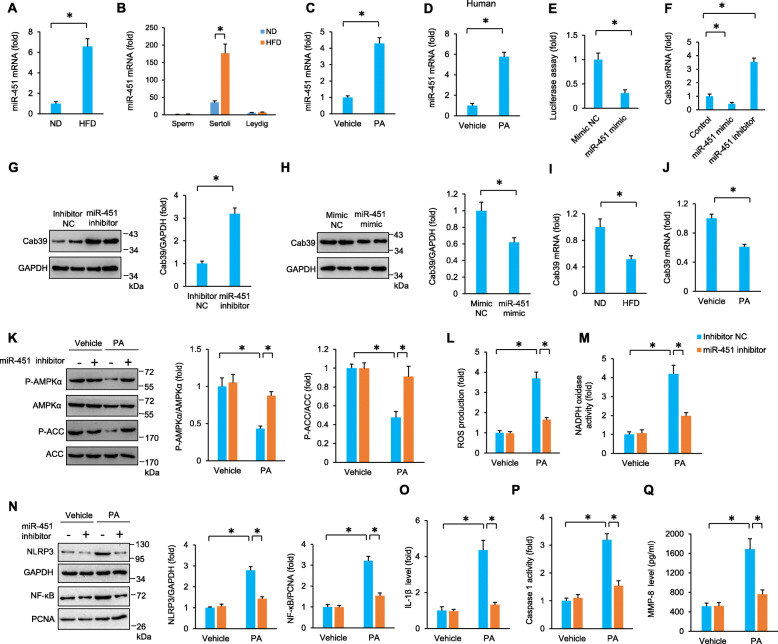
miR-451 inhibition suppressed palmitic acid-induced impairment of SCs in vitro. **A**, **B** The miR-451 level in HFD mouse testes (*n* = 6). **C**, **D** The miR-451 level in PA-treated mouse and human primary SCs (*n* = 6). **E** The luciferase assay in cultured TM4 cells (*n* = 6). **F**–**J** Cab mRNA and protein expression in cultured TM4 cells (*n* = 6). **K** The protein expression of AMPKα and ACC in PA-treated TM4 cells at 24 h after miR-451 inhibition (*n* = 6). **L**, **M** ROS production and NADPH oxidase activity in PA-treated TM4 cells at 24 h after miR-451 inhibition (*n* = 6). **N** The protein expression of NLRP3 and NF-κB in PA-treated TM4 cells at 24 h after miR-451 inhibition (*n* = 6). **O**–**Q** The IL-1β level, caspase 1 activity and MMP-8 in PA-treated TM4 cells at 24 h after miR-451 inhibition (*n* = 6). Data are presented as the mean ± SD. For **A**–**E** and **G**–**J**, the statistical analysis was carried out by Student’s two-tailed *t* test; for others, the statistical analysis was carried out by one-way ANOVA. *P < 0.05 vs the matched control

Previous studies have identified the mutual antagonism between miR-451 and AMPK signalling in regulating tumor cell migration and proliferation [32]. Therefore, we detected the expression of miR-451 in mice fed a HFD. The data in our study found that miR-451 levels were significantly increased in HFD mouse testes (Fig. 7A). Moreover, the expression pattern of miR-451 was similar to that of NLRP3. That is, obesity had no significant effect on the miR-451 levels in sperm and Leydig cells and markedly increased the miR-451 levels in SCs (Fig. 7B). We also compared miR-451 levels between SCs and testicular macrophages, and found that SCs miR-451 level was higher than that in testicular macrophages (Figure S5A). Increased miR-451 levels were also found in PA-treated mouse and human primary SCs (Fig. 7C–D). As shown in Fig. 7E, the luciferase activities were significantly decreased when miR-451 was co-overexpressed with luciferase plasmids harbouring calcium-binding protein 39 (Cab39)-3′UTR. This finding suggested that Cab39 was a target of miR-451. To confirm this, we detected the expression of Cab39 and found that Cab39 expression was downregulated by the miR-451 mimic but upregulated by a miR451 inhibitor (Fig. 7F). Cab39 protein expression was downregulated by the miR-451 mimic but upregulated by a miR-451 inhibitor (Figure S5A-B, Fig. 7G, H). Decreased Cab39 expression was also observed in obese testes and PA-treated SCs (Fig. 7I, J). Cab39 is a scaffold protein of liver kinase B1 and regulates the activation of AMPK. AMPK phosphorylation and activity were also substantially enhanced in PA-treated cells with miR-451 inhibition compared with PA-treated control mice (Fig. 7K). miR-451 inhibition also suppressed ROS production and NADPH oxidase activity in PA-treated cells (Fig 7L, M). Correspondingly, PA-induced nuclear accumulation of NF-κB, NLRP3 upregulation, IL-1β production, caspase 1 activity, and MMP-8 activity were blocked by miR-451 inhibition (Fig. 7N–Q).

### miR-451 inhibition lost protection against obesity-related impairment of spermatogenesis in AMPKα-deficient mice

**Fig. 8:**
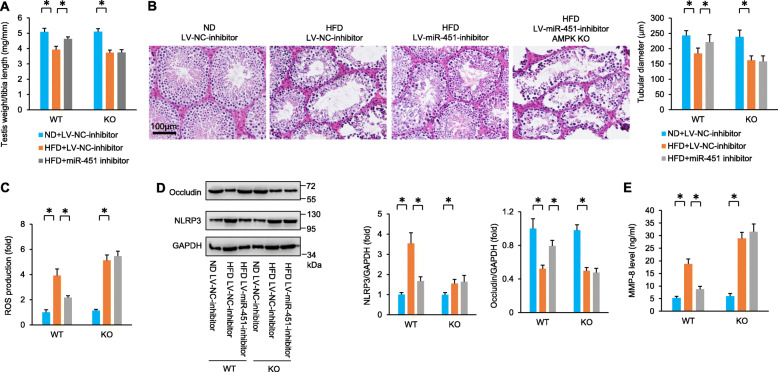
miR-451 inhibition lost protection in AMPKα-deficient mice. **A** Statistical results of the ratio of testis weight and tibia length in the indicated groups (*n* = 10). **B** HE staining of the testes and the tubular diameter from the indicated groups (*n* = 6). **C** ROS production in the testes of obese mice (*n* = 6). **D** NLRP3 and occludin protein expression in the testes of obese mice (*n* = 6). **E** MMP-8 level in the testes of obese mice (*n* = 6). Data are presented as the mean ± SD. The statistical analysis was carried out by one-way ANOVA. *P < 0.05 vs the matched control

Subsequently, we determined whether miR-451 inhibition lost its protective effects in AMPKα-deficient mice. AMPKα global knockout mice and WT littermates were subjected to an injection of lentiviral vector (LV) packing miR-451 inhibitor or NC. This lentiviral vector carrying a miR-451 inhibitor significantly decreased miR-451 expression in the testes of mice (Figure S6A). Interestingly, miR-451 inhibition did not attenuate HFD-induced impairment of spermatogenesis in the absence of AMPKα. Instead, the mice in the HFD+LV-miR-451-inhibitor + AMPK KO group exhibited a similar phenotype as those in the HFD+LV-NC-inhibitor+AMPK KO group, as reflected by the testis weight/tibia length ratio and tubular diameter (Fig. 8A, B). HFD-induced histological alterations were blocked by infection of this lentiviral vector carrying a miR-451 inhibitor, and this effect was abolished by AMPKα deficiency (Figure S6B, Fig. 8B). The HFD resulted in a decrease in sperm count, sperm viability and sperm motility, and this effect was blocked by this miR-451 inhibitor (Figure S6C). The improvement of this miR-451 inhibitor on sperm count, sperm viability and sperm motility was abolished by AMPKα deficiency (Figure S6C). miR-451 inhibition increased the testosterone and steroidogenic enzyme levels in the testes of obese mice (Figure S6D-E). However, there was no difference in testosterone and steroidogenic enzymes between the HFD + LV-miR-451-inhibitor+AMPK KO group and the HFD+LV-NC-inhibitor+AMPK KO group (Figure S6D-E). miR-451 inhibition suppressed HFD-induced ROS production, NLRP3 and MMP-8 activation, and subsequent occludin degradation in mice (Fig. 8C–E). However, these pathological effects were largely abolished by AMPKα deficiency (Fig. 8C–E).

## Discussion

The increased incidence of obesity-related spermatogenesis impairment necessitates the exploration of targets to improve therapeutic strategies. Our data provide evidence that NLRP3 promotes obesity-related spermatogenesis impairment. Using gain- and loss-of-function approaches, we found that NLRP3 promoted IL-1β secretion from SCs to impair testosterone synthesis and sperm performance and activated NF-κB and increased MMP-8 expression to degrade occludin, thus affecting the BTB and spermatogenesis. The increased miR-451 expression and subsequent AMPKα activity inhibition and ROS production were responsible for NLRP3 upregulation in the testes of obese mice. Based on the in vivo and in vitro data, we concluded that SCs NLRP3 contributes to the development of obesity-related spermatogenesis impairment. This work may provide new perspectives for developing new treatment strategies for obesity-related spermatogenesis impairment.

NLRP3 expression and functionality in mouse testes, particularly SCs, have been reported [22, 33, 34]. Moreover, NLRP3 is also expressed in the whole human testis [35]. In this study, we found that NLRP3 mRNA and protein expression were upregulated in the testes of obese mice, which was in line with a previous report where strikingly elevated NLRP3 was found in the testes of patients suffering from mixed atrophy syndrome [36]. The unique expression pattern of NLRP3 in the testes of obese mice implied a distinct role for NLRP3 during the development of obesity-related spermatogenesis impairment. In addition, previous studies reported that activated NLRP3 contributes to toxin-induced testicular hypoplasia, and ischemia-induced testicular injury in mice [21, 34, 37]. As expected, we also found that NLRP3 deficiency could prevent obesity-related spermatogenesis impairment and preserve the function of the BTB, demonstrating a maladaptive role for NLRP3 during the development of obesity-related spermatogenesis impairment. Identification of the role of NLRP3 raised another important question: which cell population is responsible for NLRP3 expression and functionality in the obesity setting? We found that NLRP3 was highly expressed in SCs and mainly derived from SCs during HFD. Using gain- and loss-of-function approaches, we found that SCs were the cell population responsible for NLRP3 expression and functionality in the obesity setting. However, we did not detect NLRP3 expression in testicular macrophage, which also played a key role in spermatogenesis impairment [38]. Li et al reported that macrophage-derived NLRP3 contributed to uropathogenic bacterial infection-induced orchitis [21]. In our future study, we will use macrophage conditional knockout mice to investigate the role of macrophage-derived NLRP3 in obesity-related spermatogenesis impairment.

Several investigations have suggested that IL-1β could repress the expression of steroidogenic enzymes in Leydig cells [21, 39]. Consistent with this finding, we also confirmed that IL-1β alone reduced testosterone synthesis. These observations raised the possibility that IL-1β secreted from SCs as a result of NLRP3 activation impaired Leydigs and sperm performance. As expected, the suppressive effects of conditioned medium collected from NLRP3-overexpressing TM4 cells on Leydigs and sperm performance were abolished with an IL-1β neutralizing antibody. These data suggested that SC-derived NLRP3 activation affected Leydig cells and sperm through IL-1β secretion.

Next, we analyzed how NLRP3-overexpressing SCs compromised the BTB. Occludin is an important component in tight junctions in the testis, and its degradation might affect cell interactions between cells, thus disrupting tight junctions and the BTB [40]. Occludin has an MMP cleavage site in extracellular loops and can be degraded by MMPs [9]. NF-κB is crucial for transcriptional regulation of MMPs and can be activated by NLRP3 activation [41]. Here, we found that NLRP3 overexpression induced nuclear NF-κB accumulation and increased MMP-8 expression. Moreover, inhibition experiments further confirmed the involvement of the NF-κB/MMP-8 axis in the degradation of occludin in NLRP3-overexpressing TM4 cells. Our finding that production of MMP8 was more specific in SCs was supported by previous studies reported the central role of MMP-8 in the disassembly of tight junction and cleavage of occludin [9, 42]. To our knowledge, this is the first report to demonstrate that NLRP3 impairs the BTB through degradation of occludin by activating the NF-κB/MMP-8 axis.

ROS have been proposed as a common signal for NLRP3 activation as most NLRP3 activators induce ROS production. Mitochondrial ROS is required for NLRP3 inflammasome activation in response to ATP [43]. In our search for triggers of NLRP3 activation in response to HFD, we focused on the production of ROS because of its key role in the activation of NLRP3 [29]. In the present study, we observed a significant increase in ROS content in the testes of obese mice along with dramatically increased NADPH oxidase activity. With the use of various ROS inhibitors, we further found that PA-induced ROS were mainly generated by NADPH oxidase. Moreover, a specific inhibitor of NADPH oxidase suppressed PA-induced NLRP3 upregulation and the degradation of occludin, which was consistent with a previous report where NADPH oxidase 4 (NOX4) promoted NLRP3 inflammasome activation via fatty acid oxidation [44]. AMPKα has been reported to suppress NADPH oxidase activity and serve as a negative regulator of ROS production [31]. Thus, we determined whether the alteration in AMPKα was responsible for ROS production. As expected, decreased AMPKα expression and activity were observed in obese testes and PA-treated cells, which was in agreement with previous studies [45, 46]. Interestingly, the restoration of AMPK activity was able to prevent SC dysfunction and NLRP3 upregulation. These data suggested that the reduced AMPKα activity and subsequent ROS production were responsible for NLRP3 upregulation in obese testes.

AMPKα and ROS also have modest expression and indispensable functionality in sperm and Leydig cells [47]. We hypothesized that there should be molecules that share a similar expression pattern with NLRP3 and regulate the activation of NLRP3. A non-coding RNA came to mind. miR-451 shared a similar expression pattern with NLRP3. Moreover, SCs miR-451 level was higher than that in testicular macrophages. These suggested that miR-451 is likely from SCs, but not from macrophages during obesity-related spermatogenesis impairment. miR-451 targeted the Cab39 protein and thus decreased AMPK expression and activity. Moreover, increased miR-451 levels were responsible for decreased AMPK phosphorylation in obese hearts [48]. Based on the in vitro and in vivo data, we found that miR-451 inhibition increased AMPK phosphorylation and suppressed ROS production, NLRP3 upregulation, and occludin degradation. These protective effects were largely abolished by AMPKα deficiency, implying the regulatory role of miR-451 in AMPKα. However, despite its important function in regulating AMPKα, there is very little known about the regulation of miR-451 in context to obesity. Ranjan et al found that ROS production might induce expression of miR-451 in macrophages [49]. That might be the reason that why obesity could induce miR-451 upregulation in mice.

## Conclusions

**Fig. 9:**
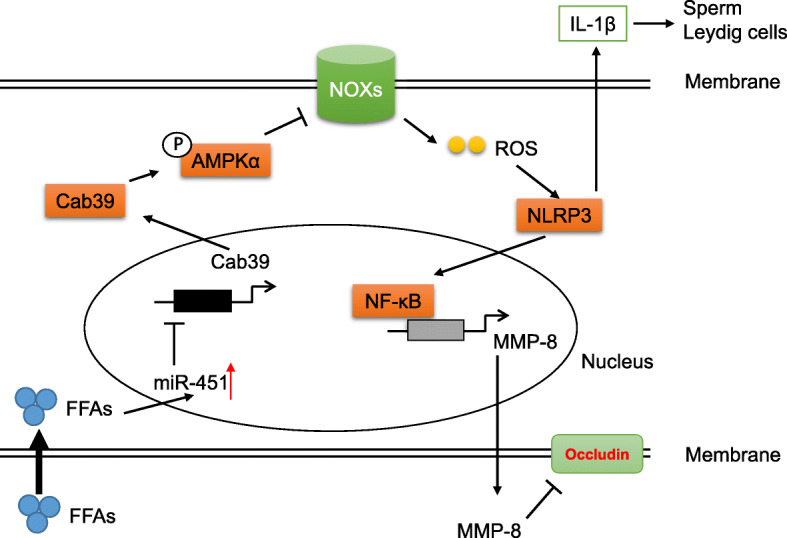
The proposed mechanism of NLRP3 in obesity-induced subfertility. Free fatty acids (FFAs) induce miR-451 expression, which impairing AMPKα activity via targeting Cab39 and thus result in NADPH oxidases-dependent ROS production. NLRP3 activation caused by excessive ROS promotes IL-1β secretion from SCs to impair testosterone synthesis and sperm performance, activates NF-κB and increases MMP-8 expression to degrade occludin, thus affecting the BTB and spermatogenesis

Our data found that NLRP3 promotes IL-1β secretion from SCs to impair testosterone synthesis and sperm performance, activates NF-κB, and increases MMP-8 expression to degrade occludin, thus affecting the BTB and spermatogenesis (Fig. 9). Targeting NLRP3 may represent a promising strategy for treating obesity-related spermatogenesis impairment.

## Materials and methods

### Animals and models

All animal studies and experiments complied with the Guidelines for the Care and Use of Laboratory Animals of the Chinese Animal Welfare Committee and were approved by the Animal Use Committees of Renmin Hospital of Wuhan University. Male C57BL mice (8 weeks) were purchased from the Institute of Laboratory Animal Science, Chinese Academy of Medical Sciences (Beijing, China). Global AMPKα2 knockout mice and global NLRP3 knockout mice were purchased from Jackson Laboratory and provided by Z. G Ma (Department of Cardiology, Renmin Hospital of Wuhan University) [31]. Heterozygous-deficient mice were interbred to establish homozygous knockout mice and their wild-type littermates in our study. These mice were bred in a specific pathogen-free facility in a temperature- and humidity-controlled environment with free access to food and water. To determine the role of NLRP3 in obesity-related spermatogenesis impairment, NLRP3-deficient mice (8 weeks old; body weight: 21–25 g) and age-matched WT littermates were fed a HFD (60% kcal, D12492, Research Diets) for 24 weeks. After a 24-week diet, the mice were sacrificed with an overdose of pentobarbital sodium (200 mg/kg), and the testis weight and tibia length were measured and recorded. Blood was obtained from the carotid artery to detect testosterone.

In another set of experiments, lentiviral vector packing a microRNA (miR)-451 inhibitor or NC was injected into mouse seminiferous tubules (2 × 10^8^ TU/mL) according to a previous study [9] at 16 weeks after HFD. This lentiviral vector carrying a miR-451 inhibitor or NC was generated by DesignGene Biotechnology (Shanghai, China). To confirm that miR-451 exerted its protection via activation of AMPKα, AMPKα global knockout mice were subjected to an injection of lentiviral vector packing a miR-451 inhibitor or NC at 16 weeks after HFD.

### Primary cell isolation, culture, and treatment

To detect NLRP3 expression and the cellular source of NLRP3 in obese testes, we isolated sperm, primary Leydig cells and SCs. The isolation of primary SCs, Leydig cells and sperm was performed according to our previous study [45]. Isolated SCs were cultured in DMEM/F12 without bovine serum albumin but containing insulin-transferrin-selenium, epidermal growth factor (2.5 ng/mL), bacitracin (5 μg/mL), and gentamicin (20 μg/mL). Insulin-transferrin-selenium (I3146) was obtained from Sigma (St Louis, MO, USA), and epidermal growth factor was obtained from Abcam (Cambridge, UK). Bacitracin and gentamicin were provided by Genaxxon Bioscience (Shanghai, China). The isolated SCs were also subjected to PA (1 mmol/L) to mimic the in vivo microenvironment of obesity as described previously [45].

To explore the role of NLRP3 in PA-induced impairment of SCs, we used TM4 cells, which were purchased from the American Type Culture Collection (Manassas, USA). This mouse cell line shares many of the characteristics of primary SCs and has been extensively used as a surrogate for primary SCs. The TM4 cells were incubated for 3 days to form tight junctions. After that, siNLRP3 or the negative control (siRNA) was transfected into the TM4 cells at a concentration of 50 nmol/L with lipofectamine RNAiMAX transfection reagent (Thermo Fisher Scientific) for 24 h. And then, this NLRP3-deficient TM4 cells were subjected to PA for 24 h. After PA incubation, this NLRP3-deficient TM4 cells were collected for the further evaluation. We also isolated SCs from NLRP3-deficient mice and WT controls, and these isolated SCs were subjected to PA incubation for 24 h. Testicular macrophages were isolated as described previously [21]. To further assess the role of NLRP3, TM4 cells were infected with adenovirus carrying NLRP3 or GFP for 6 h at a multiplicity of infection (MOI) of 50 to overexpress NLRP3 in TM4 cells [31]. Forty-eight hours after adenovirus infection, this NLRP3-overexpressed TM4 cells were collected for the further evaluation. To confirm that IL-1β in the medium of TM4 cells was responsible for the activation of NF-κB, TM4 cells with NLRP3 overexpression was incubated with a mouse IL-1β neutralizing antibody (10 μg/mL) or matched IgG for 48 h. After that, nuclear NF-κB accumulation were detected. To detect the concentration of MMP-8 in the medium released from TM4 cells, 2 days after siNLRP3 or adenovirus infection, the media were collected, and MMP-8 was detected using an MMP-8 BioAssay ELISA Kit (USBiological, Swampscott, MA). This NLRP3-overexpressed TM4 cells were also subjected to a NF-κB inhibitor (SN50, 50 μg/mL, MedChemExpress) or an MMP-8 blocking peptide (#3528RBP-50, BioVision, San Francisco, USA) for 24 h. After that, occluding and transepithelial electrical resistance in NLRP3-overexpressed TM4 cells were detected. To reveal the resource of increased cellular ROS, TM4 cells were treated with non-specific ROS inhibitors (NAC or APDC), the NADPH oxidase inhibitors (DPI or apocynin), the inhibitor of mitochondrial complex I (rotenone) and the inhibitor of mitochondrial complex II (TTFA), the endothelial nitric oxide synthase inhibitor (L-NAME) and an inhibitor of xanthine oxidase (oxypurinol) for 12 h. After that, ROS was detected in TM4 cells. The constitutively active AMPKα (caAMPKα) and GFP adenoviral vectors used in our study were constructed by DesignGene Biotechnology (Shanghai, China). To confirm the regulation of ROS production by AMPKα, TM4 cells were infected with adenovirus carrying caAMPKα at an MOI of 100 for 6 h. To explore the role of miR-451, TM4 cells were pretreated with micrON miR-451 (50 nmol/l, RiboBio Technology) or micrON mimic negative control (NC) for 48 h. To inhibit miR-451 levels, TM4 cells were pretreated with the micrOFF miR-451 inhibitor (50 nmol/l, RiboBio Technology) or micrOFF inhibitor NC.

TM3 mouse Leydig cells were purchased from the cell bank of the Chinese Academy of Sciences (Shanghai, China). The TM3 cells were cultured in DMEM/F12 medium supplemented with 10% FBS for 48 h, after which the cells were infected with siNLRP3 (50 nmol/L) or siRNA with Lipofectamine RNAiMAX transfection reagent for 24 h. After incubation with PA for 24h, TM3 cells were collected for the detection of testosterone. To disrupt testosterone production, the TM3 cells were exposed to recombinant mouse IL-1β protein (100 nmol/L, #401-ML-005, R&D Systems, USA) for 24 h. To confirm that IL-1β in the medium of TM4 cells was responsible for the impairment of TM3 cells, the conditioned medium of TM4 cells with NLRP3 overexpression was collected to stimulate the TM3 cells. The TM3 cells were cultured in dialyzed conditioned media and a mouse IL-1β neutralizing antibody (10 μg/mL) or matched IgG for 48 h. After that, testosterone production and steroidogenic enzyme expression were detected.

Mouse sperm samples were obtained from the cauda epididymis, diluted to attain a concentration of 5 × 10^6^ spermatozoa/ml and incubated in G-IVF (Vitrolife, Sweden) medium. To exclude the direct effect of NLRP3 on sperm, the sperm were incubated with an NLRP3 inhibitor (MCC950 Sodium, Selleck, 0.01 μmol/L) for 12 h, and then sperm viability and motility were assessed according to our previous studies [7, 45, 50]. These sperm samples were also subjected to recombinant IL-1β protein (100 nmol/L) for 12 h. To confirm that IL-1β in the medium of TM4 cells was responsible for the impairment of sperm, the conditioned medium of TM4 cells with NLRP3 overexpression was collected to stimulate these sperm samples in the presence of a mouse IL-1β neutralizing antibody (10 μg/mL) or the matched IgG for 12 h. After that, sperm viability and motility were assessed.

### Transepithelial electrical resistance

We used transepithelial electrical resistance across TM4 cells to reflect the integrity of tight junctions with a Millicell ERS system (Millipore, USA). TM4 cells were cultured for 3 days to form a functional tight junction barrier. After that, siNLRP3 or siRNA was transfected for 24 h. After that, the medium was changed, the TM4 cells were subjected to PA treatment for 48 h, and transepithelial electrical resistance across the TM4 cells was detected.

### Measurement of ROS generation

ROS production in the testis was determined using electron spin resonance (ESR) spectroscopy (Bruker, Karlsvuhe, Germany) with 5,5-dimetyl-1-pyrroline N-oxide (DMPO, Sigma) at a final concentration of 1 mol/L as described previously [51]. To detect ROS production in vitro, TM4 cells were incubated with 2,7-dichlorofluorescin diacetate (DCFH-DA, 10 μmol/L) for 60 min at 37 °C. The level of MDA, a product of lipid peroxidation, was measured using a commercial MDA assay kit (Abcam, ab118970).

### Real-time PCR

Testis tissue and cultured cells were lysed using TRIzol reagent, and reverse transcription was performed using the Transcriptor First Strand cDNA Synthesis Kit (Roche, Basel, Switzerland). The genes were amplified by LightCycler 480 SYBR Green 1 Master Mix (Roche) [46, 51]. The relative expression levels of the target genes were normalized to those of the controls using the 2^−△△Ct^ method. GAPDH was used as internal reference control. To detect miR-451 in the testis, we used the Bulge-Loop miRNA qRT-PCR Starter Kit (RIBOBIO, Guangzhou, China). U6 was used as an internal reference control for miRNA detection.

### Western blot

The testis tissue was lysed on ice with RIPA buffer as previously described [45, 50]. Nuclear proteins were extracted by NE-PER™ Nuclear Extraction Reagents (Thermo Scientific). The protein concentrations were measured by a BCA Protein Assay Kit. The proteins were loaded into 10% sodium dodecyl sulfate-polyacrylamide gels and transferred to PVDF membranes. The membranes were then blocked with 5% non-fat milk for 1 h and incubated with primary antibodies at 4 °C overnight and with secondary antibodies at room temperature for 1 h. Primary antibodies against NLRP3 (ab270449, 1:1000), ASC (ab151700, 1:1000), GAPDH (ab8254, 1:1000), ZO-1 (ab216880, 1:500), occludin (ab216327, 1:500), NF-κB (ab32536, 1:1000), proliferating cell nuclear antigen (PCNA, ab29), phospho-p47phox (ab63554, 1:1000), p47phox (ab166930), and Cab39 (ab51132, 1:1000) were purchased from Abcam (Cambridge, UK). Phospho-ACC (1:1000 dilution, #3661S), ACC (1:1000 dilution, #3676), phosphor-AMPK (1:1000 dilution, #2535), and AMPK (1:1000 dilution, #2603) were purchased from Cell Signalling Technology. The protein bands were detected using ECL (Bio-Rad, USA) and normalized to GAPDH.

### Luciferase reporter assay

The promoters of mouse matrix metallopeptidase (MMP)-2, MMP-8, and MMP-9 were amplified and inserted into the pGL3 basic vector. TM4 cells were cultured in 24-well plates and electrotransfected with pGL3 carrying the promotors of MMP-2, MMP-8, or MMP-9 (0.03 μg) and the pGL3-NF-κB plasmid (0.3 μg) using the Neon® Transfection System (pulse voltage: 1700 V, pulse width: 20 ms). Detection was performed using a kit according to the manufacturer’s instructions (Promega).

pMIR-REPORT™ vector was provided by Invitrogen. The pRL-TK™ Renilla reniformis luciferase plasmid was purchased from Promega. To confirm the regulation of Cab39 by miR-451, Cab39′-UTR was also inserted into the 3′-UTR of the pMIR-REPORT™ vector. After that, TM4 cells were cultured in 24-well plates and then electrotransfected with the pRL-TK™ Renilla reniformis luciferase plasmid and pMIR-REPORT™ vector.

### Testicular histopathology

Testes were collected and fixed in Bouin’s fluid for 24 h. Then, the testes were sectioned and stained with haematoxylin-eosin (H&E) to observe testicular histopathology under light microscopy (Nikon E100). The diameter of seminiferous tubules was quantified using Image-Pro Plus 6.0 software according to our previous articles [7, 45, 50].

### Immunofluorescence

Immunofluorescence of testicular tissues was performed as previously described [46]. Mouse occludin monoclonal antibody was provided by Proteintech Group, Inc. (Wuhan, China). Nuclei were stained with DAPI (Sigma, St. Louis, MO, USA). The images were observed and captured by a fluorescence microscope (Olympus, Tokyo, Japan).

### Detection of testosterone, IL-1β, MMP-8, caspase 1 activity, and NADPH oxidase activity

Fresh testis samples were homogenized to obtain the supernatant fraction. The testosterone levels and IL-1β and MMP-8 levels were quantified following the manufacturer’s protocols. Testosterone was measured using a testosterone (mouse/rat) ELISA Kit from Biovision (#K7418-100). The IL-1β levels were evaluated using a mouse IL-1β Quantikine ELISA Kit from R&D Systems (#MLB00C, USA). The MMP-8 levels were detected using an MMP-8 (Mouse) ELISA Kit from Abnova (#KA5109). The caspase 1 activity in the testes and cell extracts was measured using a Caspase 1 Colorimetric Assay Kit (#K111, BioVision, Mountain View, USA). The NADPH oxidase activity in the testes and cell extracts was determined using a commercial kit (Nanjing Institute of Jiancheng Bioengineering).

### Determination of sperm count, sperm viability, and motility

The epididymis was carefully dissected away from the fat and immediately placed into Ringer’s solution and dissected to determine the count, viability, and motility of the sperm according to previously described protocols [7, 45, 50].

### Human samples

Human studies were performed according to the Declaration of Helsinki and were approved by the human research ethics committees of Renmin Hospital of Wuhan University in Wuhan, China. The included subjects were outpatients who went to the Reproductive Medical Center of Renmin Hospital of Wuhan University for medical care between April 2018 and August 2019. They were first asked to sign an informed consent form. Then, each participant was asked to complete a questionnaire. Human testis samples were obtained by testicular puncture from men with obstructive azoospermia whose samples exhibited histologically normal spermatogenesis. Male subjects with other causes of defective spermatogenesis, including infection, varicocele, chromosomal abnormalities or smoking, were excluded from this study. Primary isolation of SCs, Leydig cells and sperm was performed as described above and according to our previous article [45]. Isolated human SCs were also subjected to PA (1 mmol/L) for 24 h.

### Data analysis

All data in this study are presented as the mean ± standard deviation (SD). Differences between two groups were compared by an unpaired *t* test. Differences in multiple groups were analyzed using one-way ANOVA with Tukey’s post hoc test using SPSS 22.0 software. *P* < 0.05 was considered significant.

## Supplementary Information

**Additional file 1: Figure S1.** NLRP3 overexpression induced NF-κB activation was largely attenuated by the IL-1β neutralizing antibody. *P< 0.05 vs the matched control. **Figure S2.** SCs-derived NLRP3 impaired testosterone production via IL-1β secretion. (A-C) The testosterone production in TM3 cells (*n*=6). (D) The mRNA levels of steroidogenic enzymes in TM3 cells after incubation with an IL-1β neutralizing antibody for 24 h (*n*=6). Data are presented as the mean ± SD. For B, statistical analysis was carried out by Student’s two-tailed t-test; for others statistical analysis was carried out by one-way ANOVA. CM: The medium collected from TM4 cells with NC; NM: The medium collected from TM4 cells with NLRP3 overexpression; *P< 0.05 vs the matched control. **Figure S3.** SCs-derived NLRP3 impaired sperm performance via IL-1β secretion. (A-B) Sperm viability and motility after NLRP3 inhibition (*n*=6). (C-D) Sperm viability and motility after IL-1β incubation for 12 hours (*n*=6). (E-F) Sperm viability and motility after IL-1β antibody treatment (*n*=6). For C-D, statistical analysis was carried out by Student’s two-tailed t-test; for others statistical analysis was carried out by one-way ANOVA. CM: The medium collected from TM4 cells with NC; NM: The medium collected from TM4 cells with NLRP3 overexpression. *P< 0.05 vs the matched control. **Figure S4.** The expression of P-ACC in TM4 cells (*n*=6). Statistical analysis was carried out by Student’s two-tailed t-test. *P<0.05 versus the matched control. **Figure S5.** miR-451 expression in several cells. (A) The expression of miR-451 in primary SCs and testicular macrophages (*n*=3-4). (B-C) The expression of miR-451 in TM4 cells (n=6). Statistical analysis was carried out by Student’s two-tailed t-test. *P<0.05 versus the matched control. **Figure S6.** miR-451 inhibition lost protection in AMPKα-deficient mice. (A) The expression of miR-451 in the testes of obese mice (*n*=6). (B) The blot of AMPK in the testes. (C) Sperm count, sperm viability and motility (*n*=6). (D) Serum testosterone production of obese mice (*n*=6). (E) The mRNA levels of steroidogenic enzymes in the testes of obese mice (*n*=6). For A, statistical analysis was carried out by Student’s two-tailed t-test; for others statistical analysis was carried out by one-way ANOVA. *P< 0.05 vs the matched control.

## Data Availability

The datasets used and/or analyzed during the current study are available from the corresponding author on reasonable request.

## Abbreviations

3β-HSD: 3β-hydroxysteroid dehydrogenase-14-15 isomerase
17β-HSD: 17β-hydroxysteroid dehydrogenase
AMPKα: AMP-activated protein kinase α
ASC: Apoptosis-associated speck-like protein
APDC: (2R,4R)-4-aminopyrrolidine-2,4-dicarboxylate
ATP: Adenosine-triphosphate
BTB: Blood-testis barrier
caAMPKα: Constitutively active AMPKα
DPI: Diphenylene iodonium
ESR: Electron spin resonance
ECL: Electrochemiluminescence
FBS: Fetal bovine serum
HFD: High-fat diet
H&E: Hematoxylin-eosin
IL-1β: Interleukin-1β
miR: MicroRNA
MMP-8: Matrix metalloproteinase-8
MOI: Multiplicity of infection
MDA: Malondialdehyde
NLRP3: Nucleotide-binding oligomerization domain-like receptor with a pyrin domain 3
NF-κB: Nuclear factor-kappa B
NADPH: Nicotinamide adenine dinucleotide phosphate
NC: Negative control
NAC: Nacetyl-L-cysteine
NOX4: NADPH oxidase 4
PA: Palmitic acid
P-ACC: Phospho-acetyl-CoA carboxylase
P450c17: 17α-hydroxylase/C17-20 lyase
ROS: Reactive oxygen species
SC: Sertoli cell
siNLRP3: SiRNA targeting NLRP3
SD: Standard deviation
StAR: Steroidogenic acute regulatory
WT: Wild type
