# Supplementary material for: Diet-induced obesity impairs spermatogenesis: the critical role of NLRP3 in Sertoli cells
Source: Inflamm Regen. 2022 Aug 2;42:24. doi: 10.1186/s41232-022-00203-z (PMC9344614; doi:10.1186/s41232-022-00203-z)
Supplement: Supplementary file 1 — Additional file 1: Figure S1. NLRP3 overexpression induced NF-κB activation was largely attenuated by the IL-1β neutralizing antibody. *P< 0.05 vs the matched control. Figure S2. SCs-derived NLRP3 impaired testosterone production via IL-1β secretion. (A-C) The testosterone production in TM3 cells (n=6). (D) The mRNA levels of steroidogenic enzymes in TM3 cells after incubation with an IL-1β neutralizing antibody for 24 h (n=6). Data are presented as the mean ± SD. For B, statistical analysis was carried out by Student’s two-tailed t-test; for others statistical analysis was carried out by one-way ANOVA. CM: The medium collected from TM4 cells with NC; NM: The medium collected from TM4 cells with NLRP3 overexpression; *P< 0.05 vs the matched control. Figure S3. SCs-derived NLRP3 impaired sperm performance via IL-1β secretion. (A-B) Sperm viability and motility after NLRP3 inhibition (n=6). (C-D) Sperm viability and motility after IL-1β incubation for 12 hours (n=6). (E-F) Sperm viability and motility after IL-1β antibody treatment (n=6). For C-D, statistical analysis was carried out by Student’s two-tailed t-test; for others statistical analysis was carried out by one-way ANOVA. CM: The medium collected from TM4 cells with NC; NM: The medium collected from TM4 cells with NLRP3 overexpression. *P< 0.05 vs the matched control. Figure S4. The expression of P-ACC in TM4 cells (n=6). Statistical analysis was carried out by Student’s two-tailed t-test. *P<0.05 versus the matched control. Figure S5. miR-451 expression in several cells. (A) The expression of miR-451 in primary SCs and testicular macrophages (n=3-4). (B-C) The expression of miR-451 in TM4 cells (n=6). Statistical analysis was carried out by Student’s two-tailed t-test. *P<0.05 versus the matched control. Figure S6. miR-451 inhibition lost protection in AMPKα-deficient mice. (A) The expression of miR-451 in the testes of obese mice (n=6). (B) The blot of AMPK in the testes. (C) Sperm count, sperm viability and [file 41232_2022_203_MOESM1_ESM.docx]

**Diet-induced obesity impairs spermatogenesis: the critical role of NLRP3 in Sertoli cells**

Yang Mu^1^, Tai-lang Yin^1^, Yan Zhang^2^, Jing Yang^1^, Yan-ting Wu^3^

^1^Reproductive Medicine Center, Renmin Hospital of Wuhan University, Wuhan 430060, China

^2^Department of Clinical Laboratory, Renmin Hospital of Wuhan University, Wuhan 430060, China

^3^Department of Reproductive Medicine, International Peace Maternity and Child Health Hospital, Shanghai Jiao Tong University, Shanghai 200030, China

**Corresponding author:**

**Yan-ting Wu**

Department of Reproductive Medicine, International Peace Maternity and Child Health Hospital, Shanghai Jiao Tong University, Shanghai 200030, China. E-mail: yanting_wu@163.com

**Jing Yang**

Reproductive Medicine Center, Renmin Hospital of Wuhan University, Wuhan 430060, China. E-mail: dryangjing@whu.edu.cn

**Yan Zhang**

Department of Clinical Laboratory, Renmin Hospital of Wuhan University, Wuhan 430060, China. E-mail: peneyyan@mail.ustc.edu.cn

**Conflict of interest**: none declared


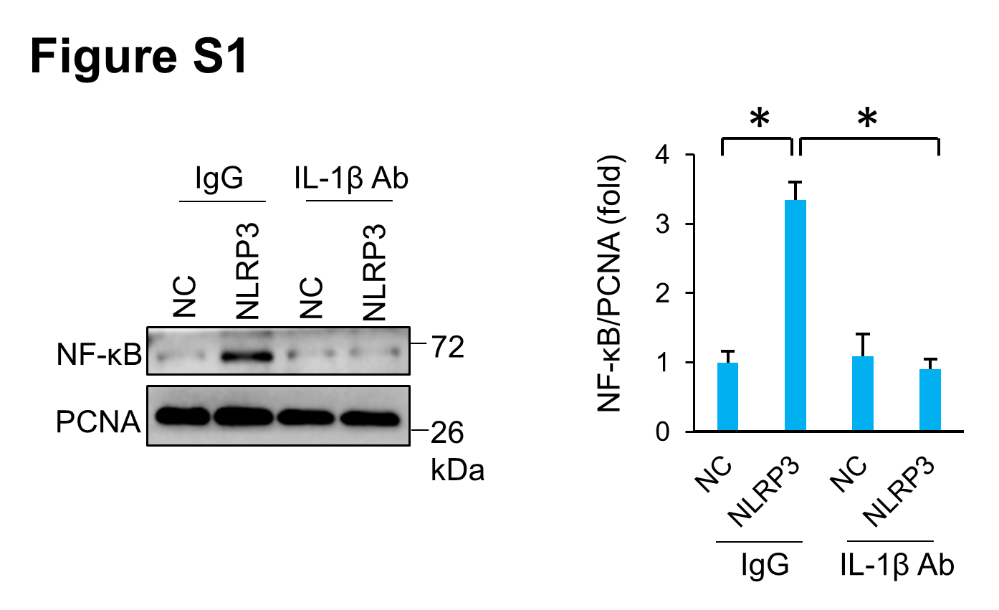


**Figure S1. NLRP3 overexpression induced NF-κB activation was largely attenuated by the IL-1β neutralizing antibody**. **P*< 0.05 vs the matched control.


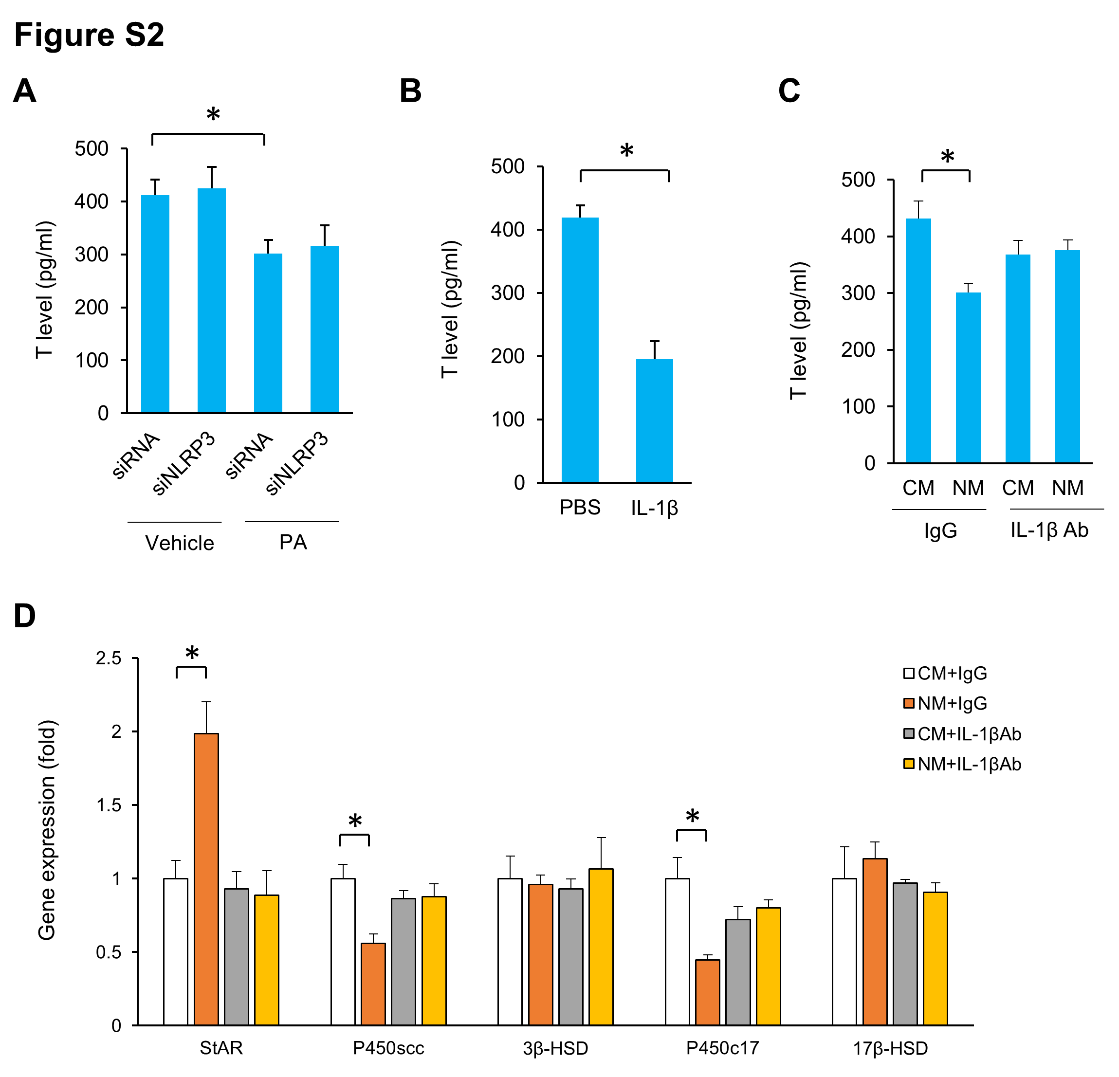


**Figure S2. SCs-derived NLRP3 impaired testosterone production via IL-1β secretion**. (A-C) The testosterone production in TM3 cells (n=6). (D) The mRNA levels of steroidogenic enzymes in TM3 cells after incubation with an IL-1β neutralizing antibody for 24 hours (n=6). Data are presented as the mean ± SD. For B, statistical analysis was carried out by Student’s two-tailed t-test; for others statistical analysis was carried out by one-way ANOVA. CM: The medium collected from TM4 cells with NC; NM: The medium collected from TM4 cells with NLRP3 overexpression; **P*< 0.05 vs the matched control.


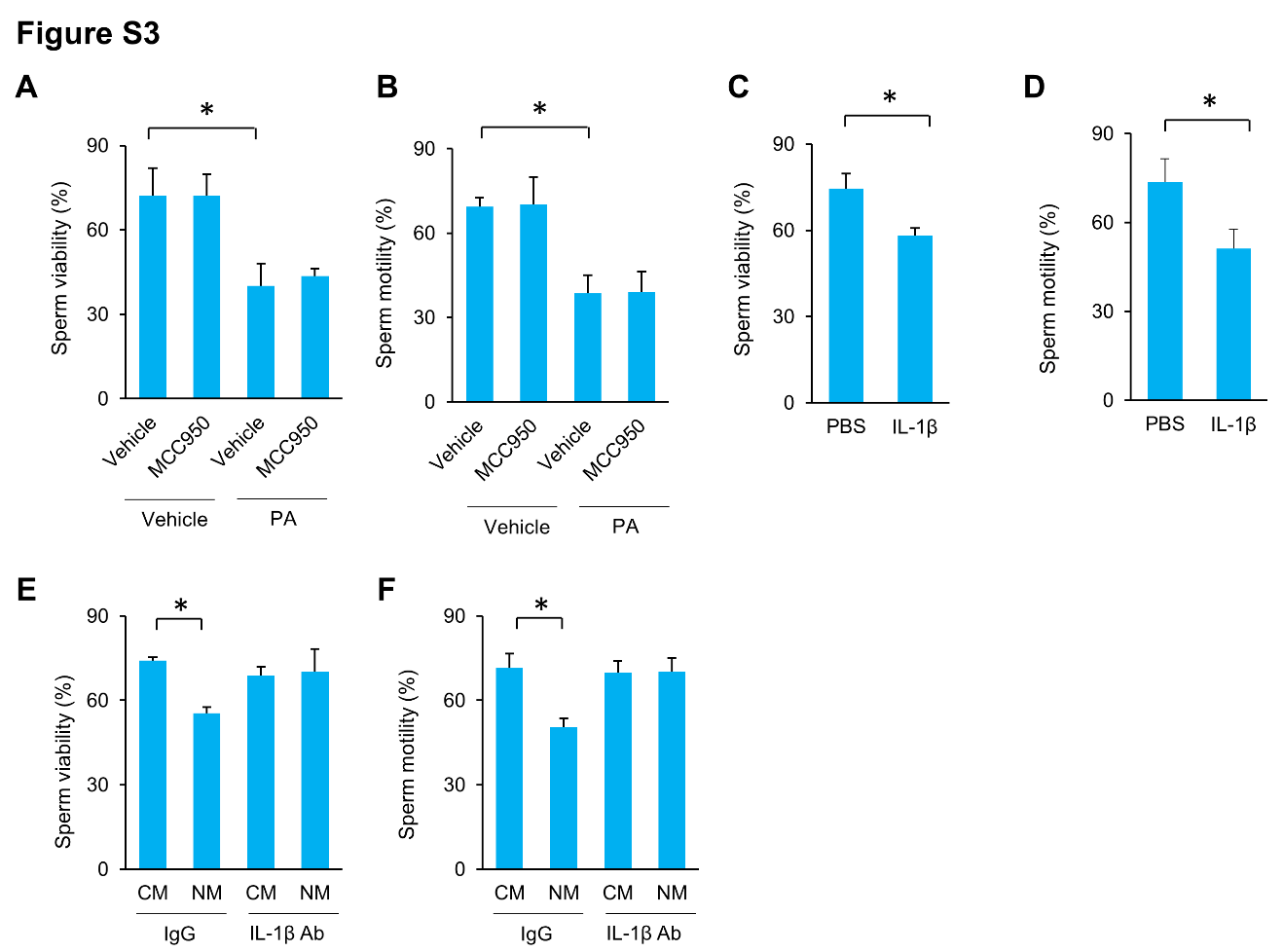


**Figure S3. SCs-derived NLRP3 impaired sperm performance via IL-1β secretion**. (A-B) Sperm viability and motility after NLRP3 inhibition (n=6). (C-D) Sperm viability and motility after IL-1β incubation for 12 hours (n=6). (E-F) Sperm viability and motility after IL-1β antibody treatment (n=6). For C-D, statistical analysis was carried out by Student’s two-tailed t-test; for others statistical analysis was carried out by one-way ANOVA. CM: The medium collected from TM4 cells with NC; NM: The medium collected from TM4 cells with NLRP3 overexpression. **P*< 0.05 vs the matched control.


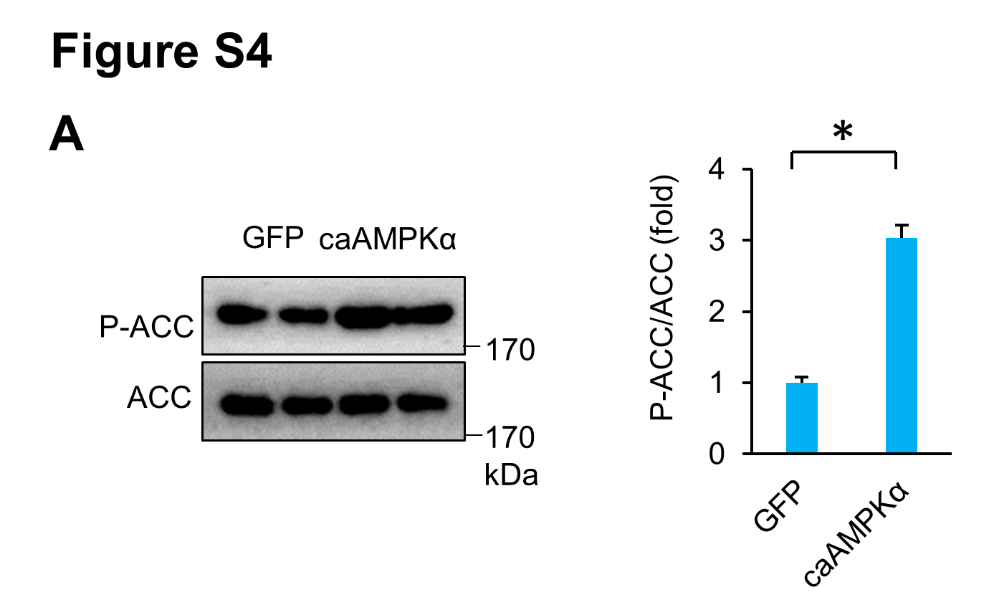


**Figure S4. The expression of P-ACC** **in TM4 cells** (n=6). Statistical analysis was carried out by Student’s two-tailed t-test. **P*<0.05 versus the matched control.


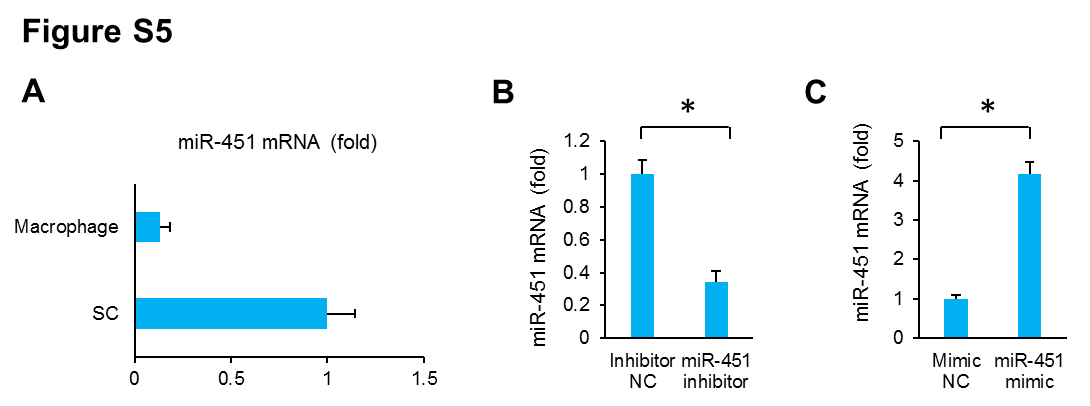


**Figure S5. miR-451 expression in** **several cells.** (A) The expression of miR-451 in primary SCs and testicular macrophages (n=3-4). (B-C) The expression of miR-451 in TM4 cells (n=6). Statistical analysis was carried out by Student’s two-tailed t-test. **P*<0.05 versus the matched control.

**
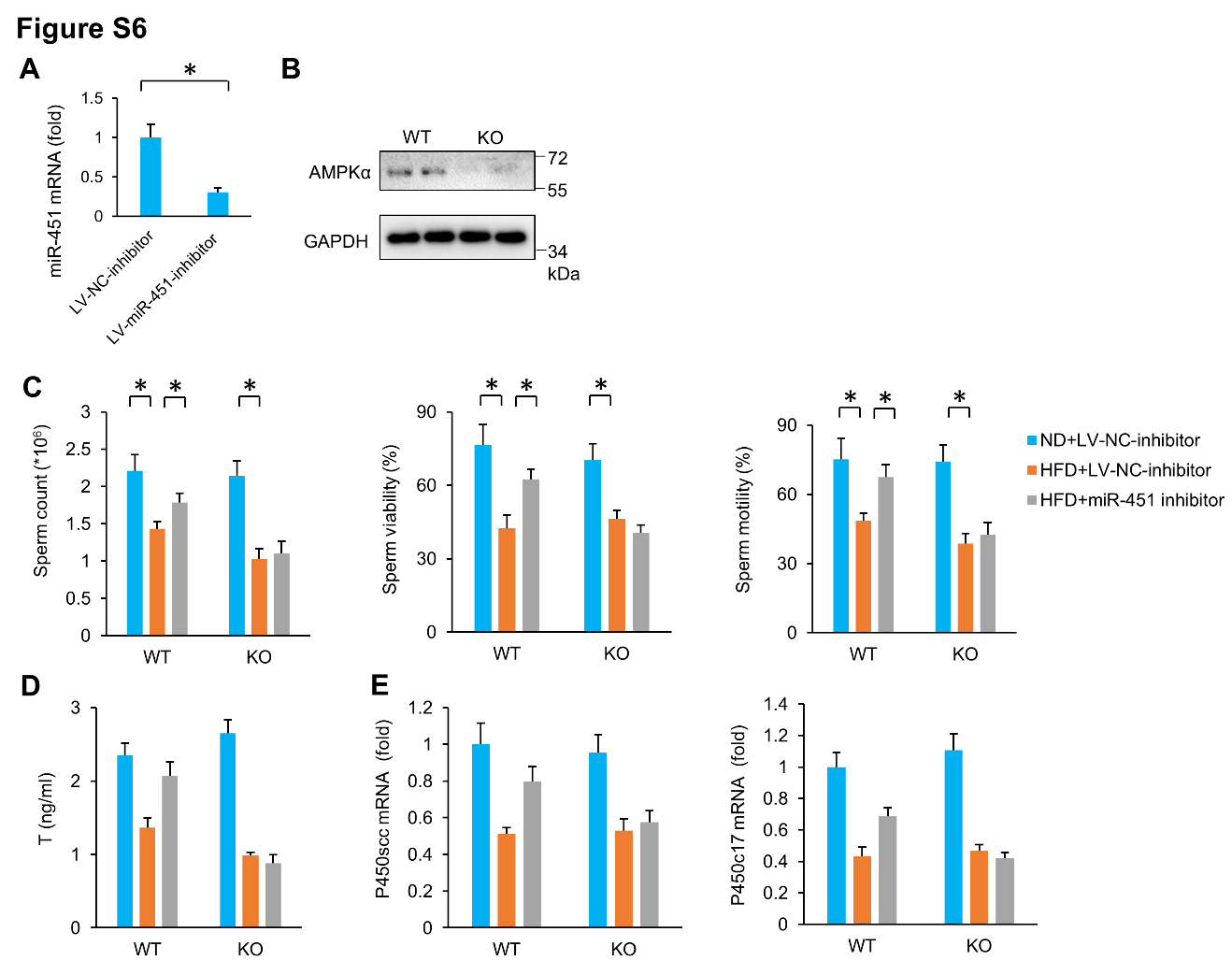
**

**Figure S6. miR-451 inhibition lost protection in AMPKα-deficient mice**. (A) The expression of miR-451 in the testes of obese mice (n=6). (B) The blot of AMPK in the testes. (C) Sperm count, sperm viability and motility (n=6). (D) Serum testosterone production of obese mice (n=6). (E) The mRNA levels of steroidogenic enzymes in the testes of obese mice (n=6). For A, statistical analysis was carried out by Student’s two-tailed t-test; for others statistical analysis was carried out by one-way ANOVA. **P*< 0.05 vs the matched control.
